# Outcomes of Transvenous Extraction of Leads Older Than 20 and 30 Years—A Large Cohort Study

**DOI:** 10.3390/ijerph192114184

**Published:** 2022-10-30

**Authors:** Andrzej Kutarski, Andrzej Głowniak, Jarosław Kosior, Wojciech Jacheć, Dorota Nowosielecka, Marek Czajkowski, Anna Polewczyk

**Affiliations:** 1 Department of Cardiology, Medical University of Lublin, 20-954 Lublin, Poland; 2 Department of Cardiology, Masovian Specialist Hospital in Radom, 20-617 Radom, Poland; 3 2nd Department of Cardiology, Silesian Medical University, 41-808 Zabrze, Poland; 4 Department of Cardiology, Pope John Paul II Hospital of Zamość, 22-400 Zamość, Poland; 5 Department of Cardiac Surgery, Medical University of Lublin, 20-954 Lublin, Poland; 6 Department of Physiology, Pathophysiology and Clinical Immunology, Collegium Medicum of Jan Kochanowski University, 25-317 Kielce, Poland; 7 Department of Cardiac Surgery, Świętokrzyskie Cardiology Center, 25-736 Kielce, Poland

**Keywords:** transvenous lead extraction, old lead extraction, safety of lead extraction, effectiveness lead extraction, old leads

## Abstract

Background: There is limited knowledge on outcome of transvenous lead extraction (TLE) of leads being 20 and 30 years old. Methods: Retrospective single center large database analysis containing 3673 TLE procedures performed from 2006 to 2020 was analysed. We aimed to compare procedure complexity and the incidence of the TLE major complications (MC) in groups where extracted leads were under 10 years, 10–20 years, 20–30 years (old) and over 30 years (very old). Results: Rate of removal of old and very old leads almost doubles with successive five-year periods (3–6-10%). In patients with old and very old leads there is an accumulation of risk factors for major complications of TLE (young age, female, multiple and/or abandoned leads, multiple previous procedures). The removal of old and very old leads was more labour-consuming, more difficult, and much more often required second-line (advanced) tools and complex techniques. Incidence of all MC grew parallel to age of removed leads from 0.6 to 18.2%; haemopericardium—from 0.3 to 12.1%, severe tricuspid valve damage—from 0.2 to 2.1%, need for rescue cardiac surgery—from 0.4 to 9.1%. Notably, there was no procedure-related death when old or very old lead was extracted. The percentages of clinical and procedural success decreased with increasing age of the removed leads from 99.2 and 97.8% to 90.9 and 81.8%. The risk of MC during extraction of leads aged 10–20 years increases 6.7 times, aged 20–30 years—14.3 times (amounting to 8.4%), and aged 30 and more years—20.4 times, amounting to 18.2%. Removal of ventricular leads is associated with a greater complexity of the procedure but not with more frequent MC. Removal of the atrial leads is associated with a higher incidence of MC, especially haemopericardium, regardless of the age of the leads, although the tendency becomes less pronounced with the oldest leads. Conclusions: 1. Extraction of old and very old leads is a rising challenge, since the rate of removal of leads aged 20-and-more years almost doubles with successive five-year periods. 2. Procedure difficulty, complexity and the risk of major complications increases along with the age of extracted lead. TLE is more time-consuming, difficult and much more often requires advanced tools and complex techniques. 3. TLE of old (≥20 years) or very old (≥30 years) leads can be performed with satisfactory success rate and safety profile when conducted at high-volume centre by an experienced operator under optimal safety conditions.

## 1. Introduction

There is a growing population of patients with CIEDs (cardiac implantable electronic devices) with a long or very long-life expectancy [1,2,3,4]. The limited, yet extending the average duration of intracardiac leads functionality (pacemaker—PM—about 20 years, implantable cardioverter-defibrillator—ICD—about 10 years) [3,4,5,6,7] and changes in the health status of patients requiring device upgrade [8,9] result in the abandonment of inactive or redundant intracardiac leads [10,11], which is accepted by successive editions of guidelines on cardiac implantable devices [12,13,14]. As a result, the population of patients with intracardiac leads, who have an expected longevity of more than 30–40 years, is growing.

Considering well-populated reports on TLE outcomes in over 1000 patients, an increasing average lead dwell time can be noticed: from 63 months in 1999–2014 to 82 months in 2018–2021 [15,16,17,18,19,20,21,22,23,24,25,26,27,28]. Despite the increase in lead dwell time, the incidence of procedure-related deaths has rather decreased. The authors of the abovementioned reports did not analyze in detail the effectiveness and safety of transvenous lead extraction of leads older than 20 and 30 years [15,16,17,18,19,20,21,22,23,24,25,26,27,28]. To the best of our knowledge, there are only two studies assessing the outcomes of lead extraction older than 30 years in 124 and 43 patients [29,30] and in a population of 16 young patients with an average age of removed lead close to 20 years [1]. In addition, the number of cases-reports regarding the removal of older leads was published [31,32,33,34,35,36,37].

Our 16 years of experience strengthened our impression that each year we remove more leads older than 20 years and that the problem of genuinely old lead extraction will grow. Limited knowledge regarding very old lead extraction encouraged us to perform detailed analysis of our TLE database including 3673 procedures. In this study, we aimed to analyse the effectiveness and safety of extraction of leads being 20 and 30 years old using mechanical systems performed by an experienced first operator in a large patient cohort of 3673 subjects. We aimed to analyse and compare procedure complexity and appearance of the major TLE complications when extracted leads were under 10 years, 10–20 years, 20–30 years and over 30 years old.

## 2. Materials and Methods

### 2.1. Study Population

All transvenous lead extraction procedures performed between March 2006 and January 2022 at a single high-volume centre were screened. Patient clinical dates, indications for TLE, CIED system and history of pacing, extracted lead information, TLE complexity, efficacy and outcomes were retrospectively analysed from our computerized database. The study population included 3673 patients (38.2% females) aged 5–97 years, average 66 year.

### 2.2. Lead Extraction Procedure

TLE indications, procedure effectiveness and complications were estimated according to recent TLE recommendations (2009 and 2017 HRS consensus and 2018 EHRA guidelines) [12,13,14]. The efficacy of TLE was expressed as the percentage of procedural success and clinical success. Procedural success was defined as the removal of all targeted leads and lead material from the vascular space with the absence of any permanently disabling complication or procedure-related death. Clinical success was defined as the removal of all targeted leads or retention of a small portion (<4 cm) of the lead that did not negatively impact the outcome goals of the procedure (i.e., residual lead did not increase the risk of perforation, embolic events, perpetuation of infection, or cause any undesired outcome) in condition of absence of any permanently disabling complication or procedure-related death [12,13,14]. Partial radiographical success was defined as the removal of all targeted leads but with retention of the tip of the lead or a non-removable small distal lead fragment (<4 cm) which did not negatively impact the outcome goals of the procedure. The complications of TLE were also defined as major complications being those that were life threatening, resulted in significant or permanent disability or death, or required surgical intervention [12,13,14].

Standard stylets or locking stylets, if needed, (Liberator Locking Stylet, Cook Medical Inc., Bloomington, IN, USA) were used for extraction of the oldest leads when estimated risk of lead break was high. Screw-out and simple traction were very rarely utilised to preserve or even rebuild the venous approach for implantation of the new or temporary pacing lead(s). First-line tool for lead dissection and extraction were non-powered mechanical telescoping polypropylene sheaths (Byrd Dilator Sheaths, Cook Medical Inc., Bloomington, IN, USA) of all sizes and lengths. The second-line tools were powered mechanical sheath systems (Evolution Mechanical Dilator Sheath, Cook Medical Inc., Bloomington, IN, USA; TightRail Rotating Dilator Sheath, Phillips Healthcare, Andover, MA, USA) or metal sheaths if the problem was located in lead venous entry and subclavian region. A combined approach, using two or more different (jugular, subclavian, femoral) access sites, was selected when conventional methods could not be effective (proximal lead ending in cardiovascular space or in case of break of extracted lead). Laser and electrosurgical dissection sheaths were not used [10,21,24,27].

Organisation of TLE procedure changed over the last 17 years from procedures performed in the electrophysiology laboratory using intravenous analgesia/sedation [21] up to procedures in the hybrid room under general anaesthesia [27]. During the last 7 years, the core extraction team has consisted of the same highly experienced TLE operator, experienced echocardiographer and dedicated cardiac surgeon [27].

Procedure complexity was expressed as procedure duration time—all lead extraction time (sheath to sheath time) and average time of single lead extraction (sheath-to sheath/number of extracted leads). The second indicator was appearance of so-called “technical problems” during TLE—situations which increased procedure complexity but not complications. Most often, lead to lead interaction or incapacity to progress with the extraction tool at the venous entry site of the leads required to use a different approach such as the femoral snaring technique [10,21,27].

### 2.3. Dataset and Statistical Methods

The Shapiro–Wilk test showed that most continuous variables were normally distributed. For uniformity, all continuous variables are presented as the mean ± standard deviation. The categorical variables are presented as number and percentage.

Four subgroups were selected for future comparative analysis. The basic criterion was the dwell time of the oldest extracted lead.

Group 1. Extracted leads under 10 years (below 120 months), 2554 patients.

Group 2. Extracted leads between 10–20 years (120–239 months), 926 patients.

Group 3. Extracted leads between 20–30 years (240–359 months), 190 patients

(old leads).

Group 4. Extracted leads over 30 years (360 and more months), 33 patients

(very old leads).

The reference point, and therefore the basic control group, was the group of patients with leads younger than 10 years (group 1).

The significance of differences between groups (2, 3, 4 vs. 1) was determined using the nonparametric Chi2 test with Yates correction or the unpaired “U” Mann–Whitney test, as appropriate. To illustrate the impact of the sum dwell time of the oldest extracted lead on survival during 30-days follow-up, the Kaplan–Meier survival curves were constructed and evaluated with log rank test. *p* < 0.05 was considered as statistical significance. Statistical analysis was performed with Statistica version 13.3 (TIBCO Software, Inc., Palo Alto, CA, USA).

### 2.4. Approval of the Bioethics Committee

All patients gave their informed written consent to undergo TLE and use anonymous data from their medical records, approved by the Bioethics Committee at the Regional Chamber of Physicians in Lublin no. 288/2018/KB/VII. The study was carried out in accordance with the ethical standards of the 1964 Declaration of Helsinki.

## 3. Results

A total of 3673 patients (38.2% female) aged 5–97 years included in our database underwent lead extraction procedures in the years 2006–2022. The rate of removal of leads with twenty-and-more years of dwell time increases with time and basically doubles with successive five-year periods, as presented in Table 1.

The characteristics of compared groups and predominant indications for TLE are presented in Table 2. Notably, the patients with old (>20 years) and very old (>30 years) leads differ from those with younger leads (<10 years and 10–20 years) at a younger age during TLE and during the first CIED implantation. The group of patients with old and very old leads is characterized by a good ejection fraction, a low percentage of ischemic disease, heart failure, and a significantly lower co-morbidity Charlson’s index. It can be said that patients with old and very old leads are young or middle-aged patients in good general health. Interestingly, the infection rate was inversely proportional to the implant duration. The analysis of the main indications for TLE showed that in patients with old and very old leads, infections, especially endocarditis, and lead dysfunction (exit/entry block, dislodgement, perforation, extracardiac pacing) was observed less frequently, but among them was much more mechanical lead damage (electric failure due to conductor fracture or insulation damage). The differences become more pronounced with the age of the leads.

The third table, construed similarly to the second one, shows system related, history of pacing related and procedure-related risk factors of major TLE complications and procedure complexity in different implant duration groups. The upper part of the table illustrates the distribution of other risk factors for major complications (apart from the age of the leads) in the compared subgroups. Patients with old (>20 years) and very old (>30 years) leads were more often implanted with a standard pacemaker, had more leads in the heart (although CRTs were rare) and, apparently, they had more previous CIED procedures. Although ICD and CS leads were very rarely removed in these patients, the number of leads removed in one patient was significantly higher, which is due to the frequent use of connected leads in these patients. The sum of the age of the removed leads, which is one of the five factors (apart from female gender, young patient age, decreased hemoglobin level and history of multiple procedures) that allow to predict the risk of major TLE complications using SAFETY TLE calculator [27], significantly increased (from 0.7% to 9.9%) across the compared groups. This indicates that in patients with old and very old leads there is an accumulation of risk factors for major complications of TLE.

The lower part of Table 3 presents TLE procedure difficulties and complexity in patients from four compared lead age groups. Procedure difficulty and complexity are represented with different indicators which value differs between the groups, growing parallelly to the extracted lead duration. Procedure duration (sheath to sheath time) rises from 10.5 to 39.0 min, average time of single lead extraction—from 6.8 to 18.2 min, frequency of so called “technical problem” during TLE (difficulty not being complications) raises from 14.3 to 54.5%, necessity to utilise venous approach other than lead venous entry from 1.6 to 12.1%, mutual lead-to-lead fusion with strong scar precluding dilatation from 3.9 to 24.2%, break of extracted lead from 1.4 to 21.2%, Byrd’s dilator collapse/deformation from 2.1 to 15.1%, block in venous lead entry region (obstructing passage) from 5.2 to 27.2%, appearance of multiple/combined mentioned difficulties from 2.2 to 33.3%. Impossibility to perform lead dilatation or appearance of mentioned technical problems required more advanced tools. The use of Evolution (old and new) or TighRail increased from 0.6 to 7.4%, metal sheath from 5.4 to 27.3%, lasso catheter/snare or basket catheter from 2.1 to 21.2%. The frequency of pacemaker dependence also raised with age of extracted leads. The table can be summarized with the statement that the removal of old (>20 years) and very old (>30 years) leads is significantly more time- and labour-consuming, more difficult, and much more often requires second-line (advanced) tools and a complex technique. The increasing age of removed leads is accompanied by an increasing frequency of other risk factors, such as abandoned leads and multiple lead extraction.

TLE outcomes in four different implant duration groups (patients with extracted leads < 10 years, 10–20 years, 20–30 years and >30 years) are presented in Table 4. Procedure outcomes most precisely represents appearance of major complications, obtained clinical and procedural success and procedure-related mortality as well. The table shows, that frequency of all major complications grows parallelly to extracted lead duration but obtained clinical and procedural success is reduced opposite extracted lead dwell time.

**Figure 1:**
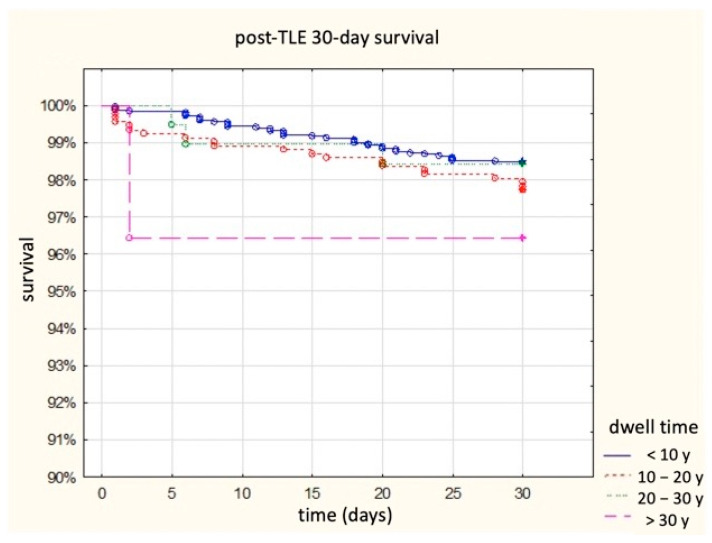
Survival after TLE in subgroups of patients depending on the sum of dwell time of the oldest extracted lead in 30-day follow-up.

Appearance of major complications (any) rises from 0.6 to 18.2%, frequency of haemopericardium increased from 0.3 to 12.1%, severe tricuspid valve damage—from 0.2 to 2.1%, necessity of rescue cardiac surgery from 0.4 to 9.1%. There was no intra-procedural death when old or very old lead were extracted. The 30-day post-procedural survival curves are presented in Figure 1. The percentages of clinical and procedural success were opposite—they decreased with increasing age of the removed leads from 99.2 and 97.8% up to 90.9 and 81.8%. The presented results can be summarized by the statement that the frequency of complications increases with the age of the removed leads, and the frequency of clinical and procedural success decreases with the age of the removed leads. The aim of the study, however, was to present the risks associated with the removal of old (over 20 years) and very old (over 30 years) intracardiac leads. The risk of lead extraction at the dwell time of 10–20 years increases 6.7 times, at the dwell time of 20–30—14.3 times to 8.4%, and at the dwell time of 30 and more—20.4 times to 18.2%.

Table 5 shows the complexity and results of TLE in the four analyzed groups, taking into account the type of the extracted leads. The purpose of the analysis presented in the table was to answer an important practical question: does the location of the lead (atrial, ventricular, both) affect the complexity, effectiveness and safety of TLE? If more than one lead needs to be extracted, start with the one that will be easier to remove; the most (potentially) dangerous lead should be removed last. In our feeling, the extraction of the old lead from the atrium is associated with an increased risk of cardiac tamponade and ventricular one—with more troublesome extraction, risk of lead rupture and leaving remnants, but not with cardiac tamponade. Common practice shows that if more leads are extracted, the risk substantially increases, though it was not studied in a large population.

The first panel of Table 5 (leads <10 years) in which the procedure complexity, major complications and TLE effectiveness were considered in the group of patients with removed “young” leads (<10 years) indicates a greater degree of procedure complexity if atrial or atrial and ventricular leads were removed during the same procedure. Twice as many major complications and haemopericardium were in the subgroup when atrial leads were removed.

The second block of Table 5 (leads 10–20 years) in which the procedure complexity, major complications and background effectiveness in the group of patients with removed leads in middle age (10–20 years) were considered, indicates a greater degree of complexity of the procedure if ventricular or atrial and ventricular lead were removed sequentially. In contrast, all major complications and haemopericardium occurred more often in the groups, atrial or atrial and ventricular leads were removed in the same patient.

The third panel of Table 5 (leads 20–30 years), in which the procedure complexity, major complications and background effectiveness in the group of patients with removed leads in advanced age was considered, shows that, as in the previous group, the greater complexity of the procedure if ventricular leads were removed or atrial and ventricular during the same procedure. Furthermore, as in the previous group, all major complications and haemopericardium occurred more often in the groups atrial or atrial and ventricular leads were removed sequentially

The fourth panel of Table 5 (leads 30 years and more), in which the procedure complexity, major complications and background effectiveness in the group of patients with the oldest leads removed, was considered, shows that in this group the differences in the complexity of the procedure between the subgroups are blurred, similarly to the previous groups all major complications and haemopericardium occurred more frequently in the groups, the atrial or atrial and ventricular leads were removed in the same patient, although the differences due to the small numbers in the subgroups were not significant. Summarizing the conclusions of Table 5, it can be stated that the removal of ventricular leads is associated with greater complexity of the procedure but not with more frequent major complications. Rather, the removal of the atrial leads is associated with a higher incidence of major complications, especially haemopericardium, regardless of the age of the leads, although the tendency becomes less pronounced with the oldest leads.

## 4. Discussion

Due to the increase of life expectancy in patients with CIED, limited lead longevity, and intentional abandonment of inactive leads accepted by HRS/EHRA guidelines [12,13,14], the number of patients with old (>20 years) and very old (>30 years) leads increases. This was confirmed by previous observations by other authors [1,29,30] and our current analyses, which showed that the rate of removal of leads older than twenty years almost doubles with successive five-year periods (3–6–10%).

An interesting observation is the increasing number of removed old leads (Table 1). There are many reasons for this, the most important of which are the greater number of patients referred for TLE and the increase in the possibility of performing the procedure (the number of CIED implantations in the EU has been almost constant for two decades, although the spectrum of devices changes, of course), the increase in survival time with CIED and a growing population with leads implanted 20 years ago or earlier that are no longer functional. It is a long-term consequence of a sharp decrease in the CIED implantation age (extension of indications) 20–25 years ago. Moreover, the number of referrals for TLE is influenced by lead management education and subsequent editions of Guidelines on Lead Management, as well as numerous publications regarding this subject.

The analysis of the elaboration of TLE results based on groups of >1000 patients shows that the mean age of the removed leads increases from 61.9 to 82.4 months (Table 6).

Table 6 shows that the increase in extracted lead dwell time was not accompanied by a change in the mean frequency of MC occurrence (1.4–2.2%) [15,16,17,18,19,20,21,22,23,24,25,26,27]. The noticeable decrease in the percentage of procedure related death should be associated with an improvement in the organization of procedures (early diagnosis of MC with TEE monitoring, general anesthesia, participation of the cardiac surgeon with the possibility of immediate emergency sternotomy). Apart from better procedural settings, we find no other explanation for this beneficial phenomenon.

Our knowledge of >20 years old lead extraction is based on 3 reports (186 patients in total) [1,29,30] and several case presentations [31,32,33]. Although previous reports have indicated that “TLE of old (≥20 years) leads can be performed with reasonable success and safety when conducted at centers with expertise in lead management” [29], the risk of old and very old leads extraction is overestimated in general opinion. Excessive worry results in the abandonment of used leads, and the problem is postponed for decades to come. Commonly held views on the removal of leads >30 years of age are not supported by any literature data.

Our analysis of 223 TLE showed that our patient population with 20- and 30-year-old leads is specific: young or middle-aged in good general health, more female, usually with pacemakers and multiple system-related risk factors (multiple/abandoned leads, many previous procedures) meaning an accumulation of risk factors for MC of TLE. A higher percentage of women and more abandoned leads have been reported by other authors [1,29,30].

Probably the young age and the prospect of long life are part of the decision to refer the patient to TLE. It is interesting that in non-infective cases mechanical lead damage (electric failure due to conductor fracture or insulation damage) dominated by a lead dysfunction (exit/entry block, dislodgement, perforation, extracardiac pacing) was observed more frequently.

The statement that the removal of very old leads is more labour-consuming, more difficult, and much more often requires second-line (advanced) tools and a complex technique is consistent with the opinion of other authors [1,29,30].

Perhaps the most interesting part of the study is the incidence of major complications. We are used to an MC cut-off of 1.4–2.2% (Table 6) [15,16,17,18,19,20,21,22,23,24,25,26,27]. However, it should be remembered that until 2017, significant damage to the tricuspid valve was not included as a separate MC [12]. Only in the guidelines of 2017 and 2018 [13,14], the damage of the tricuspid valve is listed as a separate MC. According to the cited authors, the percentage of MC removal of >20-year-old leads is 5.6% [29], 2.3% [30] and 12.5% [1]. In our material, the percentage of MC in the removal of 20–20 years of leads was 8.4%, and in the case of removal of over 30-year-old leads it was as high as 18.2%. It is worth emphasizing that among the reported 186 procedures, only one death occurred (0.54%) [1,29,30]. In our material of 223 TLE procedures, there was not a single procedure-related death.

The reported procedural success of removal of over 20-year-old leads was 90.3% [29], 94.7% [30] and 87.5% [1] (the latest study concerns very young patients). In our material of 223 TLE procedures, the procedural success of 20–30 years old leads removal was 86.8%, and 81.8% for the removal of over 30-year-old leads. This slightly low success rate was due to leaving remnants (lead fragment <4 cm or tip of lead) at 11.0%, and when removing leads over 30 years old—as high as 24.2%.

Using a bit different tools (non-powered polypropylene sheath as first line, mechanical rotational and other advanced tool as second line tools) we obtained a slightly different MC distribution: haemothorax 0% for leads >20 and >30 years old and a cardiac tamponade in 6.8% when removing >20 years old leads and 12.1% when removing >30 years old leads.

Zweiker at al. described their experience with lead extraction in 667 TLE procedures [38]. They extracted ICD leads in 34.8%, CRT-D in 33.7%, PM in 31.6% of patients and extracted lead average dwell time was 64.6 months; for ICD group 66.9—and for PM group—61.5 months. Our results, obtained in the group of patients with leads <10 years dwell time, were very similar. The slightly higher percentage of PM and CRT systems can be explained by the slower development of electrotherapy in the authors’ country 20 years ago. Patient groups with leads 10–20 years, 20–30 years and >30 years remain comparable in part only with the less numerous groups cited earlier. Notably, bearing in mind the increased risk, the cooperation of a multidisciplinary team, involving electrophysiologist and cardiac surgeon is needed, particularly with the use of rotational tools [39,40].

The analysis of the modest literature (3 reports) on the removal of old and very old leads indicates a significantly greater complexity of the procedure and, above all, a higher percentage of MC, reaching even a dozen or so percent in the case of 30-year-old leads. Beyond the obvious conclusions, there is a reflection that accompanies all “extreme” TLE procedures: “was it not possible to remove this lead 10–15 years earlier? It can be assumed that each lead in a young person with the prospect of several dozen years of life will be removed one day. Do we have to wait for class 1a indications? Does time not validate the lead abandonment strategy based on the “we do it because we can” principle?

### Study Limitations

There are several limitations of this study. Firstly, the database was prospectively integrated, but analysis was performed retrospectively. Secondly, the procedures were performed using all types of mechanical system but not laser powered sheaths. It should be noted non-powered polypropylene sheaths were first line tool when only extracted lead venous entry approach was possible. Mechanical powered rotational sheaths were used as second line tool (since were available on the country market). Needle Eye Snare and other tools were utilised when lead venous entry approach was impossible. Hence, no conclusion could be drawn on comparative efficacy or safety of mechanical non-powered versus mechanical rotational sheaths for TLE. Finally, this is the presentation of a single, very experienced first operator. It would not give an overview of general TLE safety and efficacy in patients with CIED having long and very long dwelling time. Most extractions of old or very old leads were performed in cardiac surgery operating theatre or in a hybrid room with close co-operation with a cardiac surgeon team. A certain limitation of the work, restricting the capacity to draw conclusions is the relatively small number of patients with electrodes with a dwell time of more than 30 years, although this is the largest group of such patients described so far.

## 5. Conclusions

1. Extraction of old (over 20 years) and very old (over 30 years) leads is increasing reflected by the doubling of the removal rate of such leads in successive five-year periods.
2. Procedure difficulty and complexity grows parallelly to the extracted lead age—in old leads procedures are more time-consuming, and more often requires second-line (advanced) tools and a complex technique.
3. The risk of major complications during extraction of leads 10–20 years old increases 6.7 times, 20–30 years old—14.3 times, amounting to 8.4%, and over 30 years old—20.4 times, amounting to 18.2%
4. TLE of old (>20 years) or very old (>30 years) leads can be performed with satisfactory success and safety when conducted at high volume center by experienced first operator.

## Figures and Tables

**Table 1:** Frequency of extraction of old leads (240 months and more) during following 5-years periods.

Periods (Years)	Extracted Leads Old 240 Months and More	All TLE	%
2006–2011	29	965	3.01%
2012–2016	62	1351	4.59%
2017–2022	132	1357	9.71%
All	223	3673	6.07%

**Table 2:** Patient-related risk factors of TLE complexity, major complications of TLE procedure and predominant indications for TLE (in 3673 patients).

Patient-Related Risk Factors and Predominant Indications for Tle	Leads < 10 Years		Leads 10–20 Years		Leads 20–30 Years		Leads > 30 Years		Statistic		
Number of patients/group number	2524	1	926	2	190	3	33	4	2 vs. 1	3 vs. 1	4 vs. 1
Form of results presentation (count/average; Sd/%)	Count/aver.	%/Sd	Count/aver.	%/Sd	Count/aver.	%/Sd	Count/aver.	%/Sd			
Patient’s age during TLE	66.80	14.74	64.24	17.83	63.79	15.57	65.70	11.43	*p* < 0.001	0.007	0.669
Patient’s age during first system implantation	61.90	14.97	50.57	18.01	40.72	15.46	33.00	12.04	*p* < 0.001	*p* < 0.001	*p* < 0.001
Female patients	892	35.34%	391	42.23%	102	53.68%	18	54.55%	*p* < 0.001	*p* < 0.001	0.022
Underlaying heart disease: IHD. MI	1491	59.07%	461	49.78%	82	43.16%	10	30.30%	*p* < 0.001	*p* < 0.001	0.001
NYHA III & IV	425	16.84%	108	11.66%	19	10.00%	0	0.00%	*p* < 0.001	0.014	0.004
Congestive heart failure	1223	48.46%	145	15.66%	20	10.53%	1	3.03%	*p* < 0.001	*p* < 0.001	*p* < 0.001
EF average (%)	47.24	15.62	53.77	13.57	55.62	11.81	57.73	8.71	*p* < 0.001	*p* < 0.001	*p* < 0.001
EF moderately limited (30–40%)	530	21.24%	105	11.64%	18	10.00%	1	3.70%	*p* < 0.001	*p* < 0.001	0.008
EF significantly limited (<30%)	387	15.51%	71	7.87%	8	4.44%	0	0.00%	*p* < 0.001	*p* < 0.001	0.006
Renal failure (any)	571	22.62%	154	16.63%	23	12.11%	3	9.09%	*p* < 0.001	0.001	0.090
Diabetes (any)	556	22.03%	145	15.66%	25	13.16%	5	15.15%	*p* < 0.001	0.004	0.405
Charlson’s index	5.03	3.69	4.11	3.61	3.54	3.14	3.70	3.43	*p* < 0.001	*p* < 0.001	0.039
Main indications for TLE (primary/predominant)											
Infective endocarditis with or without pocket infection	564	22.35%	211	22.79%	28	14.74%	5	15.15%	0.783	0.014	0.403
Isolated pocket infection	259	10.26%	81	8.75%	13	6.84%	0	0.00%	0.186	0.130	0.072
Mechanical lead damage *	590	23.37%	285	30.78%	96	50.53%	16	48.49%	*p* < 0.001	*p* < 0.001	0.001
Lead dysfunction **	672	26.62%	145	15.66%	8	4.21%	2	6.06%	*p* < 0.001	*p* < 0.001	0.005
Upgrading. downgrading	153	6.06%	61	6.59%	13	6.84%	3	9.09%	0.571	0.665	0.450
Abandoned lead ***	57	2.26%	35	3.78%	7	3.68%	3	9.09%	0.014	0.212	0.041
Other indications ****	279	9.07%	108	11.66%	25	13.16%	4	12.12%	0.615	0.375	0.780

* Mechanical lead damage (electric failure); ** Lead dysfunction (exit/entry block. dislodgement. perforation. extracardiac pacing); *** Abandoned lead/prevention of abandonment (AF. excessive leads); **** Other indications for TLE: MRI indication. cancer. pain of pocket. loss of indication for pacing/ICD. recapture of venous access (symptomatic occlusion. SVC syndrome. lead replacement/upgrading). threatener/potentially threatener lead (loops. free ending. left heart. LDTVD).

**Table 3:** System related, pacing related and procedure related risk factors of major TLE complications and procedure complexity in different implant duration groups.

System-Related Risk Factors of TLE Complexity and Major Complications	Leads under 10 Years		Leads 10–20 Years		Leads 20–30 Years		Leads over 30 Years		Statistic		
Number of patients/group number	2524	1	926	2	190	3	33	4	2 vs. 1	3 vs. 1	4 vs. 1
Form of results presentation	Count/aver.	%/Sd	Count/aver.	%/Sd	Count/aver.	%/Sd		Count/aver.		%/Sd	
System and history of pacing											
Pacemaker	1595	63.19%	797	86.07%	182	95.79%	33	100.0%	*p* < 0.001	*p* < 0.001	*p* < 0.001
ICD	691	27.38%	111	11.99%	6	3.16%	0	0.00%	*p* < 0.001	*p* < 0.001	*p* < 0.001
CRT-D	238	9.43%	18	1.94%	2	1.05%	9	0.00%	*p* < 0.001	*p* < 0.001	0.001
Number of leads in the heart before TLE	1.91	0.70	2.01	0.80	2.18	0.90	2.27	0.88	0.001	*p* < 0.001	0.004
≥4 leads in the heart	51	2.02%	44	4.75%	19	10.00%	3	9.09%	*p* < 0.001	*p* < 0.001	0.031
Number of procedures before TLE	1.51	0.80	2.49	1.05	3.38	1.29	4.07	1.46	*p* < 0.001	*p* < 0.001	*p* < 0.001
TLE procedure related potential risk factors of major TLE complications and procedure complexity											
ICD lead extraction	870	34.47%	126	13.61%	9	4.74%	0	0.00%	*p* < 0.001	*p* < 0.001	*p* < 0.001
CS lead extraction	211	8.36%	22	2.38%	2	1.05%	0	0.00%	*p* < 0.001	*p* < 0.001	0.107
Number of extracted leads in one patient	1.56	0.68	1.82	0.78	1.99	0.83	2.12	0.76	*p* < 0.001	*p* < 0.001	*p* < 0.001
Three or more leads were extracted	215	8.52%	125	13.50%	39	20.53%	10	30.30%	*p* < 0.001	*p* < 0.001	*p* < 0.001
Utilised approach other than lead venous entry	41	1.62%	52	5.62%	11	5.58%	2	6.06%	*p* < 0.001	*p* < 0.001	0.105
Extraction of abandoned lead(s) (any)	136	5.39%	163	17.60%	64	33.86%	15	45.46%	*p* < 0.001	*p* < 0.001	*p* < 0.001
Oldest extracted lead body dwelling time in the patient	49.44	32.20	164.1	33.81	277.2	31.98	392.5	30.26	*p* < 0.001	*p* < 0.001	*p* < 0.001
Cumulative dwell time of extracted lead (in years) in the patient	7.64	5.52	23.19	9.85	40.64	15.36	53.12	18.74	*p* < 0.001	*p* < 0.001	*p* < 0.001
Oldest extracted atrial lead dwell time	60.77	32.33	158.6	41.56	255.8	53.83	261.9	108.8	*p* < 0.001	*p* < 0.001	*p* < 0.001
Oldest extracted ventricular lead dwell time	58.20	32.15	157.1	39.62	267.5	47.66	385.1	56.27	*p* < 0.001	*p* < 0.001	*p* < 0.001
Risk of major complications (%) evaluated using SAFETY TLE calculator	0.69	0.93	3.36	3.33	7.41	6.71	9.88	8.29	*p* < 0.001	*p* < 0.001	*p* < 0.001
TLE complexity and outcomes											
Procedure duration (sheath to sheath)	10.46	14.52	22.33	30.57	35.61	37.77	39.03	30.60	*p* < 0.001	*p* < 0.001	*p* < 0.001
Average time of single lead extraction	6.81	8.96	12.34	16.46	18.31	18.33	18.30	12.92	*p* < 0.001	*p* < 0.001	*p* < 0.001
Technical problem during TLE (any) *	361	14.30%	311	33.59%	91	47.90%	18	54.55%	*p* < 0.001	*p* < 0.001	*p* < 0.001
Necessity to utilise venous approach other than lead venous entry	41	1.62%	62	6.70%	16	8.42%	4	12.12%	*p* < 0.001	*p* < 0.001	0.002
Mutual lead to lead fusion with strong scar	99	3.92%	93	10.04%	40	21.05%	8	24.24%	*p* < 0.001	*p* < 0.001	*p* < 0.001
Break of extracted lead	35	1.39%	70	7.56%	28	14.74%	7	21.21%	*p* < 0.001	*p* < 0.001	*p* < 0.001
Byrd’s dilator collapse/detorsion	53	2.10%	51	5.51%	16	8.42%	5	15.15%	*p* < 0.001	*p* < 0.001	0.001
Block in venous lead entry region	133	5.27%	100	10.80%	48	25.26%	9	27.27%	*p* < 0.001	*p* < 0.001	*p* < 0.001
Two or more technical problems	55	2.18%	68	7.34%	35	18.42%	10	30.30%	*p* < 0.001	*p* < 0.001	*p* < 0.001
Utility of additional tools											
Evolution (old and new) or TighRail	16	0.63%	24	2.59%	14	7.37%	0	0.00%	*p* < 0.001	*p* < 0.001	1.000
Metal sheath	136	5.39%	101	10.91%	48	25.26%	9	27.27%	*p* < 0.001	*p* < 0.001	*p* < 0.001
Lasso catheter/snare or basket catheter	52	2.06%	89	9.61%	30	15.79%	7	21.21%	*p* < 0.001	*p* < 0.001	*p* < 0.001
Temporary pacing during procedure	207	8.20%	147	15.87%	47	24.74%	11	33.33%	*p* < 0.001	*p* < 0.001	*p* < 0.001

* situation which increase procedure complexity but not being a complication.

**Table 4:** TLE outcomes in different implant duration groups.

TLE Outcomes	Leads under 10 Years		Leads 10–20 Years		Leads 20–30 Years		Leads over 30 Years		Statistic		
Number of patients/group number	2524	1	926	2	190	3	33	4	2 vs. 1	3 vs. 1	4 vs. 1
Form of results presentation	Count/aver.	%/Sd	Count/aver.	%/Sd	Count/aver.	%/Sd	Count/aver.	%/Sd			
TLE efficacy and complications											
Major complications	15	0.59%	37	4.00%	16	8.42%	6	18.18%	*p* < 0.001	*p* < 0.001	*p* < 0.001
Haemopericardium	7	0.28%	24	2.59%	13	6.84%	4	12.12%	*p* < 0.001	*p* < 0.001	*p* < 0.001
Haemothorax	2	0.08%	3	0.32%	0	0.00%	0	0.00%	0.124	1.000	1.000
Tricuspid valve damage during TLE (severe)	4	0.16%	10	1.08%	4	2.11%	2	6.06%	0.001	0.001	0.002
Rescue cardiac surgery	9	0.36%	22	2.38%	11	5.59%	3	9.09%	*p* < 0.001	*p* < 0.001	*p* < 0.001
Death procedure related (intra. post-procedural)	2	0.08%	4	0.43%	0	0.00%	0	0.00%	0.048	1.000	1.000
Death indication-related (intra. post-procedural	1	0.04%	2	0.22%	1	0.53%	0	0.00%	0.177	0.135	1.000
30-ty daysmortality	38	1.51%	21	2.27%	3	1.58%	1	3.03%	0.167	0.819	0.996
Partial radiographic success	46	1.82%	71	7.67%	21	11.05%	8	24.24%	*p* < 0.001	*p* < 0.001	*p* < 0.001
Full clinical success	2503	99.17%	882	95.25%	180	94.74%	30	90.91%	*p* < 0.001	*p* < 0.001	0.003
Full procedural success	2469	97.82%	840	90.71%	165	86.84%	27	81.82%	*p* < 0.001	*p* < 0.001	*p* < 0.001

**Table 5:** TLE complexity and outcome in four analysed groups taking into accounts kind of extracted leads.

**TLE Complexity and Outcomes**	**Ventricular Lead Extracted Only**		**Atrial Lead Extracted Only**		**Extracted Atrial and Ventricular Leads**		**Statistic**	
Number of patients/group number	1134	1	300	2	1090	3	2 vs. 1	3 vs. 1
Form of results presentation	Count/aver.	%/Sd	Count/aver.	%/Sd	Count/aver.	%/Sd		
Extracted leads with dwell time under 10 years (119 and less months)								
Oldest extracted lead body dwelling time	56.37	32.06	63.25	33.14	61.57	31.83	0.001	*p* < 0.001
Number of extracted leads in one patient	1.06	2.25	1.17	0.41	2.20	0.51	0.370	*p* < 0.001
Extraction of abandoned lead(s) (any)	38	3.35%	8	2.67%	90	8.26%	0.550	*p* < 0.001
Single lead extraction time ****	7.19	10.14	7.97	10.80	6.11	6.79	0.243	0.003
Technical problem during TLE (any) *	130	11.46%	42	14.00%	189	17.34%	0.229	*p* < 0.001
Two or more technical problems	12	1.06%	3	1.00%	40	3.67%	1.000	*p* < 0.001
Utility of second line (advanced) tools **	74	10.53%	48	16.00%	152	13.96%	*p* < 0.001	*p* < 0.001
Major complications (any)	5	0.44%	4	1.33%	6	0.55%	0.098	0.770
Haemopericardium	0	0.00%	3	1.00%	4	0.37%	0.009	0.058
Procedure-related death (intra-/post-procedural)	0	0.00%	0	0.00%	2	0.18%	1.000	0.240
Lead remnants ***	13	1.15%	7	2.33%	26	2.39%	0.119	0.026
Full clinical success	1130	99.65%	300	100.0%	1073	98.44%	0.586	0.004
Full procedural success	1117	98.50%	293	97.67%	1059	97.16%	0.317	0.029
Extracted leads with dwell time between 10–20 years (120–239 months)								
Number of patients/group number	265	1	136	2	525	3	2 vs. 1	3 vs. 1
Oldest extracted lead body dwelling time	159.6	31.24	169.6	35.86	164.9	34.39	0.004	0.036
Number of extracted leads in one patient	1.13	0.36	1.42	0.57	2.27	0.65	*p* < 0.001	*p* < 0.001
Extraction of abandoned lead(s) (any)	41	15.47%	18	13.23%	104	19.81%	0.549	0.137
Single lead extraction time ****	15.92	23.82	9.52	10.03	11.26	12.52	0.003	*p* < 0.001
Technical problem during TLE (any) *	78	29.43%	34	25.00%	199	37.91%	0.349	0.018
Two or more technical problems	13	4.91%	4	2.94%	51	9.71%	0.440	0.019
Utility of second line (advanced) tools **	70	26.42%	21	15.44%	123	23.43%	0.013	0.356
Major complications (any)	4	1.52%	5	3.68%	28	5.33%	0.174	0.012
Haemopericardium	2	0.76%	3	2.21%	19	3.62%	0.342	0.018
Procedure-related death (intra-/post-procedural)	0	0.00%	1	0.74%	3	0.57%	0.339	0.555
Lead remnants ***	14	5.28%	8	5.88%	49	9.33%	0.803	0.047
Full clinical success	261	98.49%	130	95.59%	491	93.52%	0.095	0.001
Full procedural success	248	93.59%	126	92.65%	466	88.76%	0.723	0.030
Extracted leads with dwell time between 20–30 years (240–359 months)								
Number of patients/group number	52	1	20	2	118	3	2 vs. 1	3 vs. 1
Oldest extracted lead body dwelling time	282.0	36.98	276.0	29.11	275.2	30.07	0.528	0.211
Number of extracted leads in one patient	1.33	0.51	1.350	0.49	2.39	0.73	0.864	*p* < 0.001
Extraction of abandoned lead(s) (any)	19	36.54%	2	10.00%	43	36.75%	0.041	0.990
Single lead extraction time ****	21.99	19.27	12.90	11.51	17.61	18.64	0.053	0.165
Technical problem during TLE (any) *	25	48.08%	5	25.00%	61	51.70%	0.110	0.664
Two or more technical problems	3	5.77%	0	0.00%	32	27.12%	0.555	0.001
Utility of second line (advanced) tools **	24	46.15%	5	25.00%	63	53.39%	0.117	0.384
Major complications (any)	3	5.77%	1	5.00%	12	10.17%	1.000	0.558
Haemopericardium	2	3.85%	1	5.00%	10	8.48%	1.000	0.348
Procedure-related death (intra-/post-procedural)	0	0.00%	0	0.00%	0	0.00%	1.000	1.000
Lead remnants ***	8	15.39%	0	0.00%	13	11.02%	0.096	0.425
Full clinical success	49	94.23%	20	100.0%	111	94.07%	0.555	1.000
Full procedural success	43	82.69%	20	100.0%	102	86.44%	0.055	0.525
Extracted leads with dwell time >30 years (360 and more months)								
Number of patients/group number	10	1	2	2	21	3	2 vs. 1	3 vs. 1
Oldest extracted lead body dwelling time in the patient	381.5	17.41	367.5	6.36	400.1	33.81	0.303	0.114
Number of extracted leads in one patient	1.20	0.42	2.00	110.0	2.57	0.68	0.027	*p* < 0.001
Extraction of abandoned lead(s) (any)	4	4.00%	1	50.00%	10	47.62%	1.000	1.000
Single lead extraction time ****	18.50	13.44	12.50	13.43	18.75	13.29	0.577	0.961
Technical problem during TLE (any) *	6	60.00%	1	50.00%	11	52.38%	1.000	1.000
Two or more technical Problems	1	10.00%	1	50.00%	8	38.10%	0.318	0.205
Utility of second line (advanced) tools **	6	60.00%	1	50.00%	9	42.86%	1.000	0.458
Major complications (any)	1	10.00%	1	50.00%	4	19.05%	0.318	1.000
Haemopericardium	0	0.00%	1	50.00%	3	14.29%	0.167	0.533
Procedure-related death (intra-/post-procedural)	0	0.00%	0	0.00%	0	0.00%	1.000	1.000
Lead remnants ***	4	40.00%	0	0.00%	4	19.05%	0.515	0.381
Full clinical success	8	80.00%	2	100.0%	20	95.24%	1.000	0.237
Full procedural success	7	70.00%	2	100.0%	18	85.71%	1.000	0.358

* Technical problems during TLE: necessity to utilise venous approach other than lead venous entry; mutual lead-to-lead fusion with strong scar; break of extracted lead; Byrd’s dilator collapse/detorsion and block in venous lead entry region; ** Utility of second line tools: Evolution (old and new) or TighRail; metal sheath; lasso catheter/snare; basket catheter; *** Lead remnants means partial radiological success (remained tip or <4 cm lead fragment); **** Single lead extraction time (sheath-to-sheath/number of extracted leads).

**Table 6:** Review of large reports (over 1000 TLE procedures) in aspects of mean implant duration and frequency of major complications in 4 following periods.

Year, Author, Journal [Ref]	Type of the Study	Number of pts	First Line Tool	Implant Duration (Months)	% of Infective Indications	Major Complications	Procedure Related Death
Studies 1999–2014							
1999 Byrd CL, Pacing Clin Electrophysiol [15]	U.S. Extraction Database analysis	2338	Cook’s extraction kit *	47	27.00%	1.40%	0.40%
2008 Bongiorni M, Eur Heart J [16]	Single-centre study	1193	Cook’s extraction kit *	69	82.00%	0.70%	0.30%
2010. Wazani O, JACC [17]	Multi-centre register	1449	Laser sheath	82	57.00%	1.40%	0.30%
2014 Brunner MP, HeartRhythm [18]	Single-centre study	2999	Laser 70%	61	43.00%	1.80%	0.20%
All studies 1999–2014		7979		61.9	46.69%	1.45%	0.29%
Studies 2015–2017							
2016 Bashir J, Circ Arrhythm Electrophysiol [19]	The British Columbia Cardiac Registry	1082	Laser	129	45.00%	3.00%	0.37%
2017 Hussein AA, JACC Clin Electrophysiol [20]	Single-centre study	1836	Laser. Evolution as second	107.5	100.00%	1.93%	0.29%
2017 Kutarski A, Europace [21]	Single-centre study	2049	97% Cook’s extraction kit *	89	40.00%	1.80%	0.36%
All studies 2017–2018		4967		104.6	63.27%	2.11%	0.34%
Studies 2017–2018							
2017 Bongiorni M, Eur Heart Journal [22]	The European Lead Extraction ConTRolled Registry (ELECTRa)	3555	Laser 19.3%	76.8	52.00%	1.70%	0.50%
2018 Sood N, Circ Arrhythm Electrophysiol [23]	Multicenter register	11,304	Laser 63%	65	14.00%	2.30%	0.16%
All registers 2018–2021		14,859		67.8	23.09%	2.16%	0.24%
Studies 2018–2021							
2019 Jacheć W, Pacing Clin Electrophysiol. [10]	Two-centres study	3810	98% Cook’s extraction kit *	86.4	46.10%	1.44%	0.17%
2020 Segreti L, Europace [25]	Single-centre study	1210	Cook’s extraction kit *	72	67.00%	0.70%	0.16%
2020 Starck CT, Europace [26]	Multicenter study (PROMET)	2205	rotational TLE tools	74	46.00%	1.00%	0.18%
2020 Giannotti Santoro M, Pacing Clin Electrophysiol [27]	Single-centre study	1316	Cook’s extraction kit *	72	65.70%	0.70%	0.00%
2021 Stefańczyk P, Vasc Health Risk Manag [28]	Single-centre study	1000	Cook’s extraction kit *	112	22.00%	2.20%	0.00%
All studies 2018–2021		9541		82.4	48.90%	1.22%	0.13%
ALL studies 1999–2021		37,346		75.2	40.07%	1.76%	0.24%

* Cook’s extraction kit: looking stylets. dilator sheaths. and/or transfemoral approach using snares; retrieval baskets; sheaths and if necessary—other tools.

## Data Availability

The data supporting reported results of the study can be accessed at www.usuwanieelektrod.pl.
